# Identification of Foxm1 as a critical regulator for metabolic dysfunction-associated steatotic liver disease by epigenomic and transcriptional profiling

**DOI:** 10.1016/j.cellin.2026.100325

**Published:** 2026-04-14

**Authors:** Chuanfei Zeng, Mingliang Wei, Huan Li, Fengyuan Niu, Ziqing Guo, Lian-Yun Li, Min Wu, Ming-Kai Chen

**Affiliations:** a Department of Gastroenterology, Renmin Hospital of Wuhan University, Wuhan University, Wuhan, 430072, Hubei, China; b State Key Laboratory of Metabolism and Regulation in Complex Organisms, Frontier Science Center for Immunology and Metabolism, Hubei Key Laboratory of Cell Homeostasis, Hubei Key Laboratory of Developmentally Originated Disease, College of Life Sciences, Taikang Center for Life and Medical Sciences, Renmin Hospital of Wuhan University, Wuhan University, Wuhan, 430072, Hubei, China; c Zhangshan School of Medicine, Sun Yat-Sen University, Guangzhou, 510080, Guangdong, China; d Guangzhou National Laboratory, Guangzhou, 510005, Guangdong, China

**Keywords:** MASLD, Epigenomics, Enhancer, Foxm1, Lipid metabolism

## Abstract

Epigenetic regulation has emerged as a key mechanism in metabolic dysfunction-associated steatotic liver disease (MASLD). However, the systematic epigenomic profiling for MASLD progression is still lacking. To investigate the epigenetic mechanisms regulating MASLD, this study performed chromatin immunoprecipitation sequencing (ChIP-Seq) for H3K27ac, H3K4me1, H3K4me3, H3K9me3, and H3K27me3, along with transcriptomic profiling, using liver tissues from multiple stages of a Gubra-Amylin NASH (GAN) diet-induced mouse model. Transcriptomic analysis defined the 8- and 16-week time points as the inflammation stage, and the 20- and 24-week as the fibrosis stage. Chromatin state analysis revealed that enhancer and polycomb regions increase during MASLD progression. Differential enhancers were defined based on H3K27ac peaks, and Foxm1 was identified as a key transcription factor involved in MASLD. *In vitro* and *in vivo* experiments demonstrate that lipid droplets accumulate in *Foxm1*-knockdown liver cells. Further studies indicate Foxm1 represses MASLD progression by regulating key genes involved in lipid storage and cholesterol homeostasis. Taken together, our work has provided important datasets and identified Foxm1 as a repressive transcription factor for MASLD progression.

## Highlights

• The temporal epigenomic dynamics is profiled in the GAN-diet MASLD model.
• Enhancer and polycomb regions increase during MASLD progression.
• Foxm1 is a key transcription factor in MASLD, whose protein level decreases along with disease progression.
• Foxm1 represses MASLD by regulating key genes involved in lipid storage and cholesterol homeostasis.

## Graphical abstract

**Figure:**
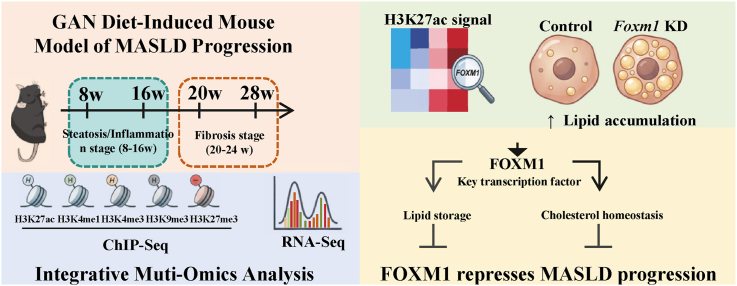

## 1 Introduction

Metabolic dysfunction-associated steatotic liver disease (MASLD), formerly termed as non-alcoholic fatty liver disease (NAFLD), is a metabolic disorder characterized by excessive hepatic fat accumulation. It is closely linked to obesity, diabetes, insulin resistance, and metabolic syndrome. Globally, the prevalence of MASLD from 1990 to 2019 has been estimated at approximately 30% (Le et al., 2022). Currently, it is one of the fastest-growing causes of liver-related mortality worldwide and has imposed a substantial public health and economic burden on societies (Paik et al., 2020). Among patients, 3%–15% of those with MASH progress to cirrhosis, while 4%–27% of those with MASH-related cirrhosis develop hepatocellular carcinoma (HCC) (Dhamija et al., 2019). The pathogenesis of MASLD, including its subsequent progression to MASH and cirrhosis, results from complex interactions involving hepatocytes and extrahepatic organs. Many factors have been shown to contribute to MASLD development, including insulin resistance, oxidative stress, chronic inflammation, hepatic stellate cell activation, dysregulated cytokine and adipocytokine signaling, genetic predisposition, and epigenetic modifications (Marra and Svegliati-Baroni, 2018; Streba et al., 2015). Several promising drugs are under clinical investigation, but combination regimens are probably required due to the complexity of the disease. Currently, healthy lifestyle interventions and weight reduction remain the cornerstone of MASLD prevention and management (Powell et al., 2021).

Epigenetic factors, including DNA methylation, histone modifications, chromatin remodeling, and non-coding RNA (ncRNA), play critical roles in metabolic diseases, cancer and inflammation (Lee et al., 2014; Sodum et al., 2021; Waddington, 2012; Wu et al., 2023). Aberrant modifications have been reported in the pathogenesis of insulin resistance and subsequent MASLD development (Loomba et al., 2021). The prevalent modification H3K27ac, enriched at active promoters and enhancers, is critical for the transcriptional programming in many biological processes, including in hepatic inflammatory and MASLD progression (Becares et al., 2019; Chen et al., 2022; Li et al., 2021; Lin et al., 2023; Smith and Shilatifard, 2014). H3K4me1, linked to gene activation, marks regulatory regions and correlates with open chromatin structures at enhancers, while H3K4me3 is associated with active transcription and mainly distributed at transcription start sites. KMT2D/MLL4, a methylase for H3K4, promotes *Idi1* expression by catalyzing H3K4me1 deposition at its promoter, thereby impairing exercise-induced amelioration of MASLD (Fan et al., 2024). Similarly, the MLL4 complex mediates H3K4me1/2 modifications to critically upregulate Ccl2 expression in hepatocytes and M1 macrophage marker genes (Lee et al., 2024). Conversely, reduced H3K4me3 on lipogenic gene promoters decreased lipogenesis and macrophage activation, ultimately improving MASH pathology (Luo et al., 2024). H3K9me3 is a hallmark of heterochromatin and represses gene transcription. Studies in MASLD mouse models have revealed reduced peak number and intensity in H3K9me3-marked genomic regions (Zhang et al., 2023b), with enhancers at these loci implicated in dysregulated lipid metabolism and inflammatory pathways. Aberrant H3K9me3 deposition at *PPARα* (peroxisome proliferator activated receptor alpha) and lipid catabolism network genes contributes to lipid accumulation (Jun et al., 2012). H3K9me3 removal from LXR response elements (LXREs) is crucial for LXRα-mediated lipogenic programming, ultimately promoting hepatic steatosis (Kim et al., 2020). Removal of H3K27me3, which is associated with gene silencing and chromatin compaction, leads to target gene upregulation (Cai et al., 2021). However, systematic research on the dynamic distribution of histone modifications during MASLD progression is still lacking.

To comprehensively understand the epigenomic dynamics during MASLD progression, the current study profiled the distribution of multiple histone modifications and performed chromatin state analysis in a high-fat, high-cholesterol, high-fructose (Gubra-Amylin NASH, GAN) diet-induced MASLD mouse model. The GAN diet is recognized as a gold standard model mimicking human NASH/MASH pathology, including hepatic steatosis, inflammation, ballooning, and fibrosis, thereby providing an ideal platform for in-depth mechanistic exploration of epigenetic regulation (Mollerhoj et al., 2022; Nielsen et al., 2023; Vacca et al., 2024). Together, this study provides important datasets for understanding epigenetic and transcriptional regulation and identifies novel biomarkers and therapeutic targets in MASLD.

## 2 Results

### 2.1 Comprehensive multi-omics profiling of mouse liver samples

**Fig. 1:**
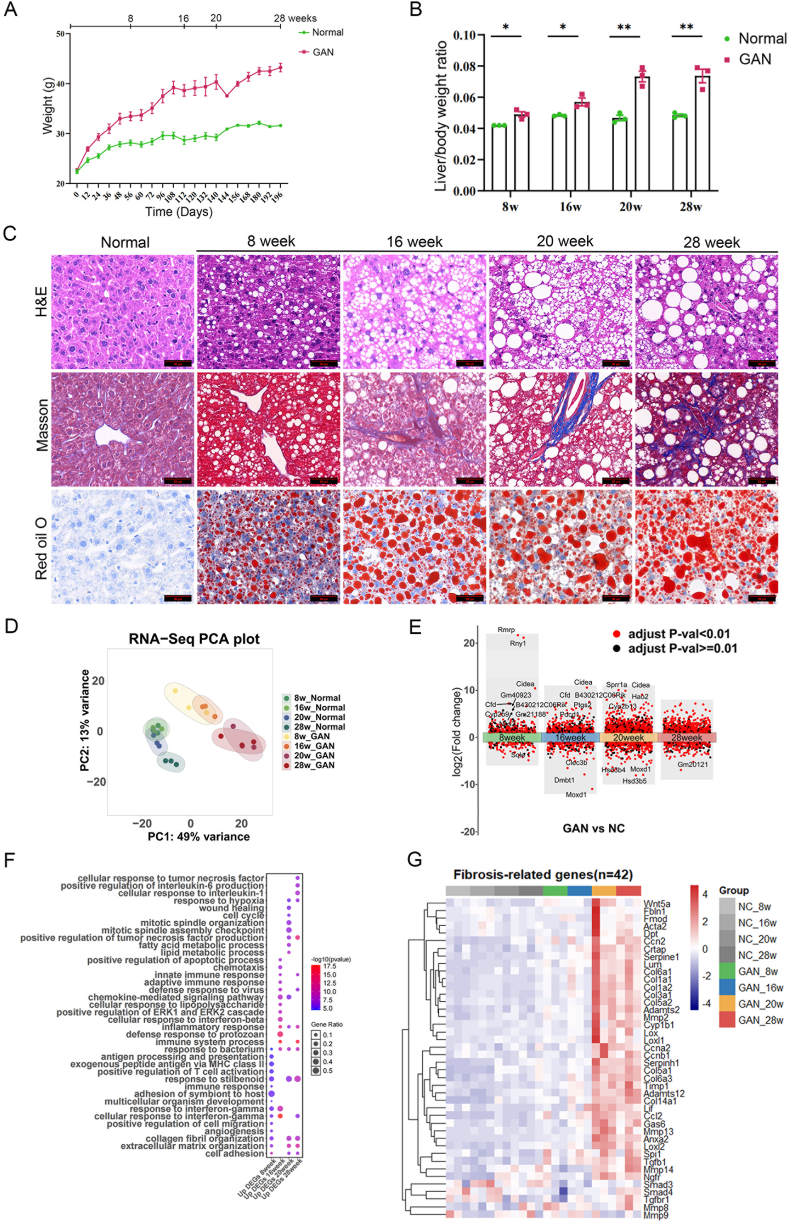
**Characterization of MASLD progression and multi-omics profiling in GAN diet-fed mice. (A)** Body weight changes in GAN diet-induced MASLD mouse models at 8, 16, 20, and 28 weeks. **(B)** Ratio of liver weight to body weight in mice at 8, 16, 20, and 28 weeks. **(C)** Representative liver H&E, Masson, and Oil Red O staining in mice at 8, 16, 20, and 28 weeks. Scale bar = 50 μm. **(D)** PCA results of RNA-Seq data. **(E)** Volcano plot showed differentially expressed genes (DEGs) between the control and GAN-fed groups at 8, 16, 20, 28 weeks. Genes with |fold change| ≥ 2 and adjusted *P*-value (*P*adj) ≤ 0.01 were highlighted in red. Non-significant genes were shown in black. **(F)** GO functional analysis of up-regulated DEGs in the livers of MASLD mice. **(G)** Heat maps of fibrosis-related genes in the liver of MASLD mice at 8, 16, 20, and 28 weeks.

Male C57BL/6J mice (5 weeks old) were randomly assigned to either Normal group or GAN group. The Normal group was fed a standard maintenance diet, while the GAN group received a high-fat, high-cholesterol, high-fructose diet. Based on the literatures, MASLD mouse models induced by dietary manipulation exhibit characteristic pathological changes over time. By 8 weeks of high-fat diet feeding, significant hepatic steatosis is typically observed; around 16 weeks, features of steatohepatitis become evident; after 20 weeks, fibrosis begins to develop and progressively advances (Ma et al., 2025a; Van Eyck et al., 2024). Mice were sacrificed at designated time points: 8, 16, 20 and 28 weeks. Compared to the Normal group, mice in the GAN group exhibited significantly increased body weight and liver-to-body weight ratio (Fig. 1(A) and (B)). Pathological staining revealed that the GAN group developed mild steatosis at 8 weeks, more severe steatosis with mild ballooning at 16 weeks, severe steatosis with inflammatory infiltration at 20 weeks, and severe steatosis, inflammatory infiltration, as well as fibrosis at 28 weeks (Fig. 1(C)). Glucose tolerance tests (GTT) showed impaired glucose tolerance in all four groups of MASLD mice (Fig. S1A–D), indicating a reduced ability of the organism to metabolize glucose. Serum AST and ALT levels, reflecting liver injury, progressively increased with prolonged GAN diet feeding (Fig. S1E and F). Serum TC, hepatic TC, and hepatic TG content were significantly elevated in the GAN group at all four time points, while serum TG showed no significant difference in the GAN group (Fig. S1G–M). In summary, our GAN diet-induced MASLD mouse model recapitulated the progression pattern of MASLD and was suitable for subsequent analyses.

### 2.2 Epigenomic and transcriptomic analysis of MASLD livers

Subsequently, the liver tissues of the Normal group (*n* = 3) and the GAN group (*n* = 3) were selected at 8, 16, 20, and 28 weeks for RNA-Seq analysis. PCA results showed that biological replicate samples within the same experimental group clustered closely in the PCA space, indicating high biological consistency in the data. Samples from the Normal groups at all four time points almost overlapped and were significantly separated from samples from the GAN groups, demonstrating substantial gene expression differences caused by the different treatment conditions. Furthermore, within the GAN group, the 20 and 28-week time points could be clearly distinguished from the 8-week and 16-week time points (Fig. 1(D)). Gene function analysis showed that the differentially expressed genes (DEGs) at 8 and 16 weeks were enriched in inflammatory response and metabolic pathways; and those at 20 and 28 weeks were enriched in inflammatory response, metabolism, wound healing and collagen fibril organization (Fig. 1(E–G), Fig. S2). These indicate that the 8- and 16-week time points were likely at the inflammatory stage, while the 20- and 28-week time points were at the fibrotic stage.

**Fig. 2:**
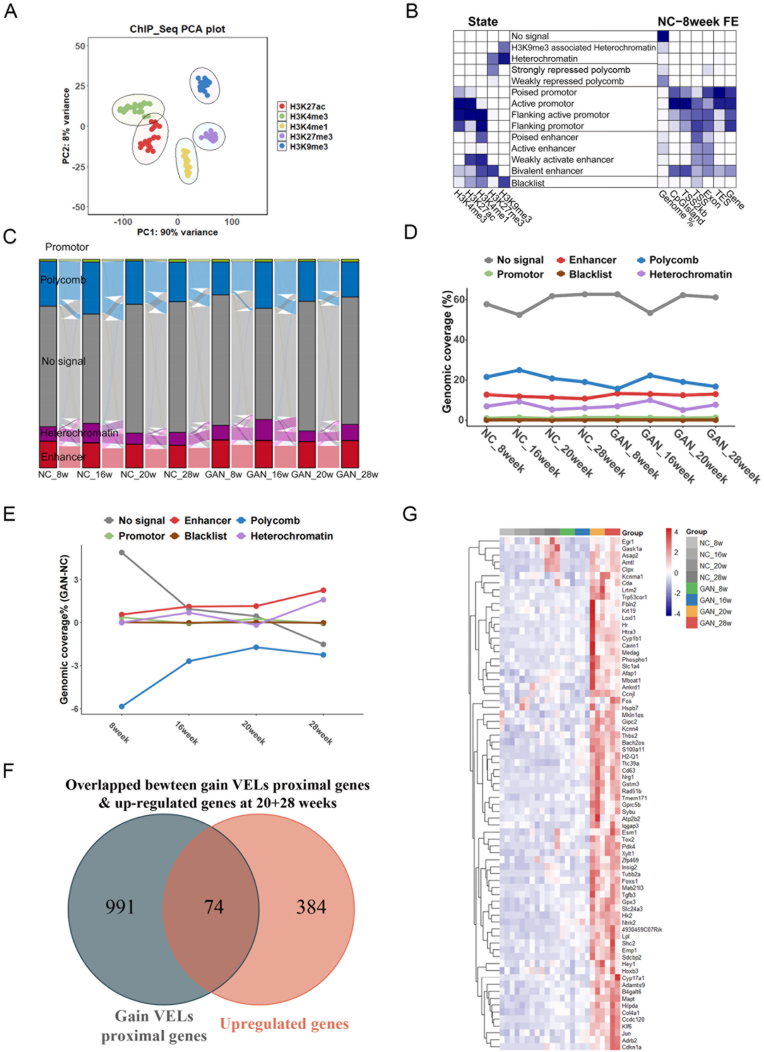
**Dynamic changes of chromatin state during MASLD progression. (A)** PCA results of ChIP-Seq data. **(B)** The definitions of chromatin states were based on the enrichment of histone modifications, which was determined using the ChromHMM software. The genome was partitioned into 14 chromatin states, and these states were subsequently grouped into six major types: No signal, Heterochromatin, Polycomb, Promoter, Enhancer, and Black list region. The right panel shows chromatin state enrichments on various genomic elements. FE means fold enrichments. **(C)** Sankey diagram to show the dynamic changes of five principal chromatin states across different time points of GAN diet feeding. **(D)** Line graph to show the genomic occupancy of six chromatin states across different developmental time points. **(E)** The bar plot displayed the differences in genomic coverage (%) of six chromatin states between GAN diet and normal diet mice at four time points (8, 16, 20, and 28 weeks). Positive values (GAN > NC) represented an increase in genomic coverage upon GAN diet feeding, while negative values (GAN < NC) represented a decrease. **(F)** Venn diagram to illustrate the overlap between genes proximal to fibrosis-stage (20 and 28 week) gained enhancers and up-regulated genes. **(G)** The heatmap shows the expression levels of the 74 overlapping genes (F) in eight samples. Expression values were *Z*-score normalized based on FPKM.

To investigate the epigenomic dynamics of MASLD, ChIP-Seq analyses of H3K4me3, H3K27ac, H3K4me1, H3K27me3 and H3K9me3 with the above tissues were carried out. PCA results of the ChIP-Seq data showed that the samples of the same histone modifications were grouped together (Fig. 2(A)). The active histone modifications, H3K4me1, H3K27ac, and H3K4me3 exhibited higher correlation across different samples, while H3K27me3 and H3K9me3 separated from the active modifications (Fig. S3A–B).

**Fig. 3:**
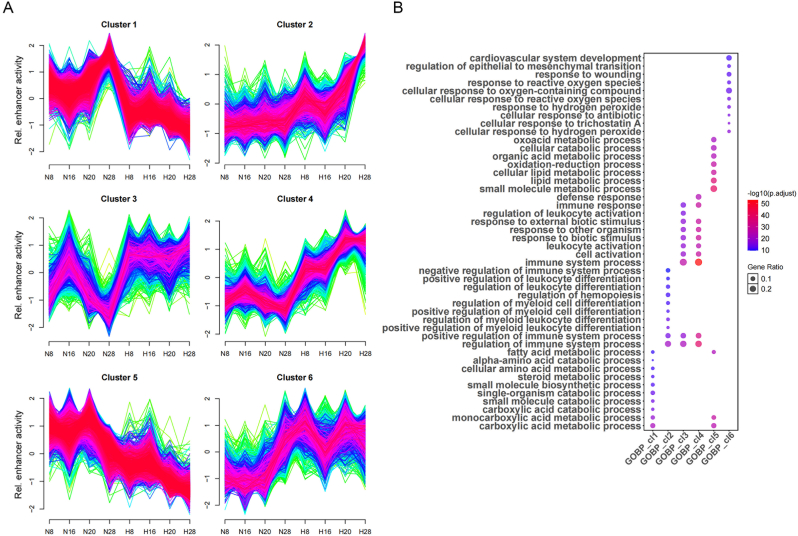
**Temporal dynamics of enhancer activity in MASLD progression. (A)** Mfuzz time-series clustering of H3K27ac-marked variant enhancer loci (VELs) in Normal and GAN groups at 8, 16, 20 and 28 weeks identified six clusters. **(B)** GO analysis of the genes in the above six clusters.

### 2.3 Dynamic chromatin states regulated by histone modifications

To study the dynamic chromatin states during MASLD progression, we utilized the software ChromHMM to analyze the ChIP-Seq dataset. We classified the chromatin regions into 14 types according to the combinatory patterns of histone modifications, including no signal, H3K9me3 associated chromatin, heterochromatin, strongly repressed polycomb, weakly repressed polycomb, poised promoter, active promoter, flanking active promoter, flanking promoter, poised enhancer, active enhancer, weakly activated enhancer, bivalent enhancer, and blacklist regions (Fig. 2(B)). The 12 types, excluding no signal and blacklist regions, could be categorized into 4 main regions, heterochromatin, polycomb, promoter and enhancer regions (Fig. 2(B), Tables S2–4). Analyses of all four chromatin regions indicated that they were all dynamically changed at different stages (Fig. 2(C–E), Fig. S3C–I). The comparison between GAN groups and control groups at different time points showed that the genome coverage of enhancer regions and polycomb regions continuously increased along with disease progression (Fig. 2(E)). The overlap of the upregulated DEGs and the target genes of gained variant enhancer loci (VELs) at the 28-week stage revealed 74 genes, which might be involved in MASLD progression (Fig. 2(F) and (G)). The temporal expression pattern of these genes aligned closely with disease progression; therefore, they may play important roles in the transition from the steatosis/inflammation stage to the fibrotic stage.

### 2.4 Identification of key pathways for MASLD progression by temporal analysis of enhancer activity

The previous analyses revealed the importance of dynamic enhancers in MASLD progression. Temporal dynamics analysis may further clarify the roles of enhancers in the regulation of biological processes (Chen et al., 2022). We then utilized the software Mfuzz and identified six distinct enhancer clusters (Fig. 3(A), Table S5). Clusters 1 and 5 showed continuously decreased expression and were mainly enriched in lipid metabolism pathways; clusters 2, 3 and 4 exhibited sustained or increased activity associated with leukocyte differentiation and activation, indicating metabolic reprogramming and immune hyperactivation drive disease progression (Fig. 3(B)). Cluster 6 was enriched with oxidative stress and epithelial to mesenchymal transition, marking pro-fibrotic transformation with impaired regeneration (Fig. 3(B)).

Parallel gene expression profiling via MaSigPro also revealed six dynamic patterns (Fig. S4A–G, Table S6). Cluster 1 demonstrated persistently low expression in cholesterol metabolism, indicating early metabolic impairment. Cluster 2 showed transient early upregulation in innate immunity, suggesting immune activation-exhaustion transition. Cluster 3 displayed progressive increase in inflammatory/hypoxic responses, promoting chronic injury and fibrogenesis. Cluster 4 maintained suppression of lipid/energy metabolism pathways, reflecting worsening metabolic dysfunction. Clusters 5 and 6 exhibited late-stage activation in lipid synthesis/cell proliferation and fibrosis/matrix remodeling respectively, indicating concurrent compensatory hyperplasia and pathological fibrosis advancement. Taken together, the temporal analyses of dynamic enhancers and transcriptomics revealed similar but not identical transcriptional programs in MASLD progression. It may gain novel insights by integrating the above data.

### 2.5 Reprogramming of heterochromatic H3K9me3 during MASLD progression

H3K9me3 is a repressive epigenetic mark associated with gene silencing and heterochromatin formation. In MASLD mouse models, the number of H3K9me3 loss sites increased progressively, peaking at 28 weeks (Fig. S5A). Functional enrichment analysis revealed that early loss (8 weeks) was concentrated in cholesterol biosynthesis and other lipid metabolism processes. As the disease advanced, enriched pathways shifted to signal transduction (16 weeks), nucleophagy and the Wnt pathway (20 weeks), and ultimately widespread dysregulation of transcriptional control, cell differentiation, and material transport (28 weeks) (Fig. S5B). At 28 weeks, H3K9me3 loss was accompanied by substantial activation of transposable elements. The overall proportion of transposable elements increased significantly (Fig. S5C), with prominent rises in LTR, LINE, and simple repeat elements. Among these, LTR elements constituted the largest fraction, reaching up to 41.9% of all activated elements ( Fig. S5D). Further classification highlighted ERVK and ERV1 as the major LTR subfamilies derepressed under H3K9me3 deficiency. At 28 weeks, 1 075 enhancer sites with H3K9me3 loss were identified, characterized by decreased H3K9me3 and increased H3K27ac signals (Fig. S5E). Functional analysis indicated that these sites were associated with cytokine production, insulin response, cell cycle regulation, and catabolic processes. These findings suggest that H3K9me3 loss may drive MASLD progression toward fibrosis and severe liver injury by promoting inflammatory responses, exacerbating insulin resistance, and disrupting cell proliferation and lipid metabolism balance (Fig. S5F).

### 2.6 Identification of Foxm1 as one key regulator via differential enhancer analysis

**Fig. 4:**
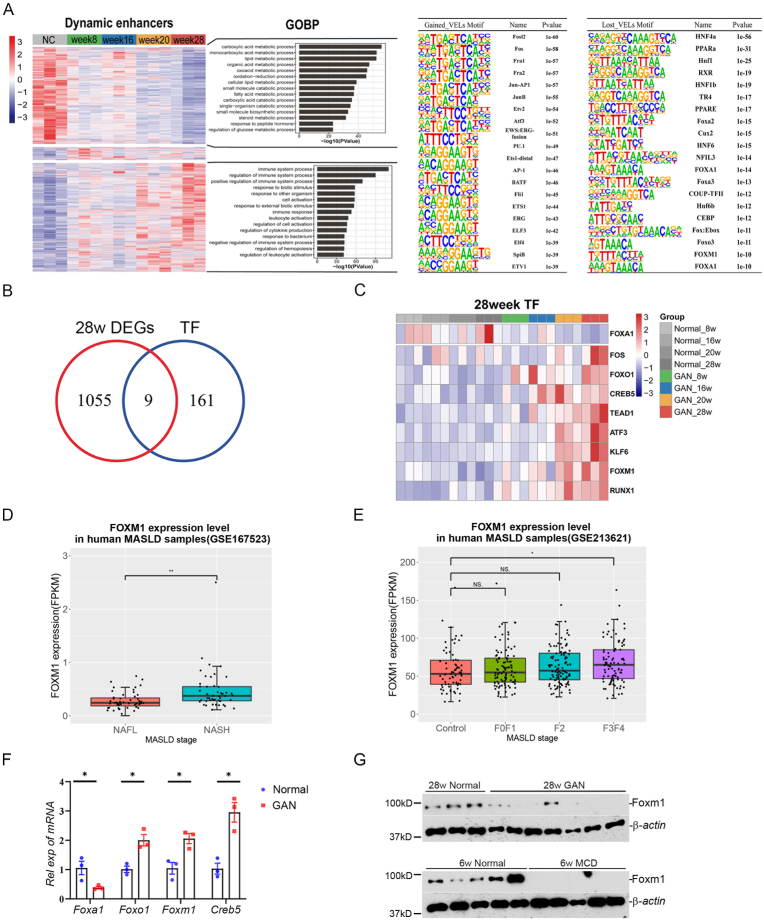
**Identification of Foxm1 as a key transcription factor in MASLD. (A)** Heatmap to show the activity of all dynamic enhancers in five stages. Expression values were *Z*-score normalized based on RPKM. The control groups (8, 16, 20, 28 weeks) merged into an NC group. Bar plot to show the top significantly enriched GO terms for the clustered enhancers groups (Left). Intersection analysis of transcription factors (TFs) predicted from hepatic VELs and DEGs in 28-week MASLD mice (Right). **(B)** Venn diagram to illustrate the overlap between DEGs and TFs in 28-week MASLD mice. **(C)** Heatmap of gene expression for the 9 identified transcription factors in MASLD mice. **(D&E)** Foxm1 expression in human liver transcriptome datasets (GSE167523 and GSE213621). **(F)** Hepatic mRNA levels of *Foxa1*, *Foxo1*, *Foxm1*, and *Creb5* in 28-week MASLD mice. **(G)** Hepatic Foxm1 protein expression in MASLD mice induced by 28-week GAN diet and 6-week MCD diet.

The information of differential enhancers can be used to predict key transcription factors (TFs) using motif analysis. The up-regulated and down-regulated enhancers at 28-week was used for TF prediction (Fig. 4(A)). The predicted TFs were then overlapped with the DEGs at 28-week, and we obtained 9 candidates (Fig. 4(B)). Among them, FOXA1 was down-regulated, and all others showed increased expression during MASLD progression (Fig. 4(C)). The roles of FOXA1, FOXO1, FOS, KLF6, RUNX1 and TEAD1 in MASLD have been reported, supporting the accuracy of our analyses (Bechmann et al., 2013; Hsiao et al., 2024; Kaur et al., 2019; Kim et al., 2025; Pang et al., 2025; Sommerauer et al., 2024). It was interesting that three members of Forkhead transcription factor family were involved in the disease, and the function of Foxm1 had not been studied, then we focused on Foxm1 in the following study. Analysis of public datasets shows *Foxm1* mRNA level increase during MASLD progression (Fig. 4(D) and (E)). Experimental validation found that the mRNA of *Foxa1* decreased in the livers of GAN-diet mice, while the mRNA of *Foxm1*, *Foxo1* and *Creb5* increased (Fig. 4(F)). Interestingly, Foxm1 protein level decreased in the livers of GAN-diet mice and MCD (methionine-choline-deficient)-diet mice, suggesting that Foxm1 participated in MASLD and post-translational regulation was involved (Fig. 4(G)).

### 2.7 Foxm1 represses steatosis in MCD-diet model

**Fig. 5:**
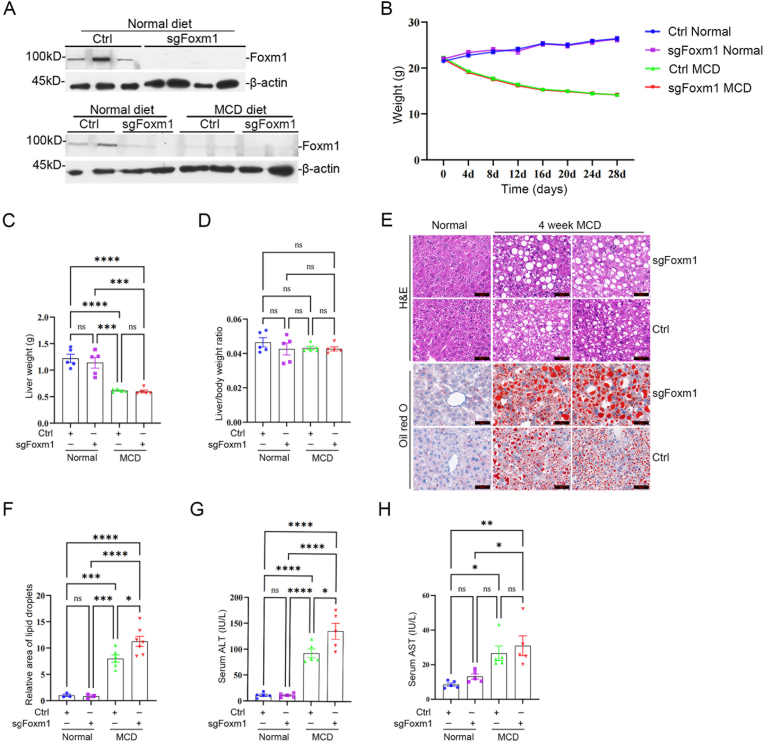
**Foxm1 represses steatosis in MASLD livers. (A)** Western blot analysis of Foxm1 protein levels in the indicated groups. **(B**–**H)** Body weight (B), Liver weight (C), Liver-to-body weight ratio (D), Liver H&E and Oil Red O staining (E), Statistical analysis of hepatic lipid droplet content (F), Serum ALT (G) and Serum AST (H) levels in the control and sgFoxm1 mice. Scale bar = 50 μm. ∗*P* < 0.05, ∗∗*P* < 0.01, ∗∗∗*P* < 0.001, ∗∗∗∗*P* < 0.000 1; ns indicates not significant.

To investigate the function of Foxm1 in MASLD, we knocked out Foxm1 by hydrodynamic injection of Cas9 and sgFoxm1 plasmids into tail veins, and fed the mice with MCD diets to induce MASLD. The MCD diet rapidly induces hepatic steatosis and inflammation within a short timeframe (2-4 weeks) without the confounding metabolic factors such as obesity and insulin resistance, making it well-suited for validating gene functions in lipid metabolism and early inflammatory regulation (Cui et al., 2025; Larter and Yeh, 2008; Zhang et al., 2024). This combined approach, using the GAN diet for mechanistic discovery and the MCD diet for functional validation, ensures both clinical relevance and experimental efficiency. Western blot results indicated that Foxm1 decreased in the livers of MCD-diet mice, and Foxm1 was successfully knocked out in both normal and MCD-diet livers (Fig. 5(A)). Compared to the control mice fed with an MCD diet, the Foxm1-deficient mice on the MCD diet showed no significant differences in body weight, liver weight, or liver/body weight ratio (Fig. 5(B–D)). Foxm1 deficiency markedly exacerbated the lipid deposition induced by MCD diet in liver cells (Fig. 5(E) and (F)). Serum ALT levels were significantly increased, and AST levels exhibited an upward trend (Fig. 5(G) and (H)). These indicated that Foxm1 represses MASLD pathogenesis by regulating lipid metabolism.

### 2.8 Foxm1 regulates lipid metabolism in liver cells

**Fig. 6:**
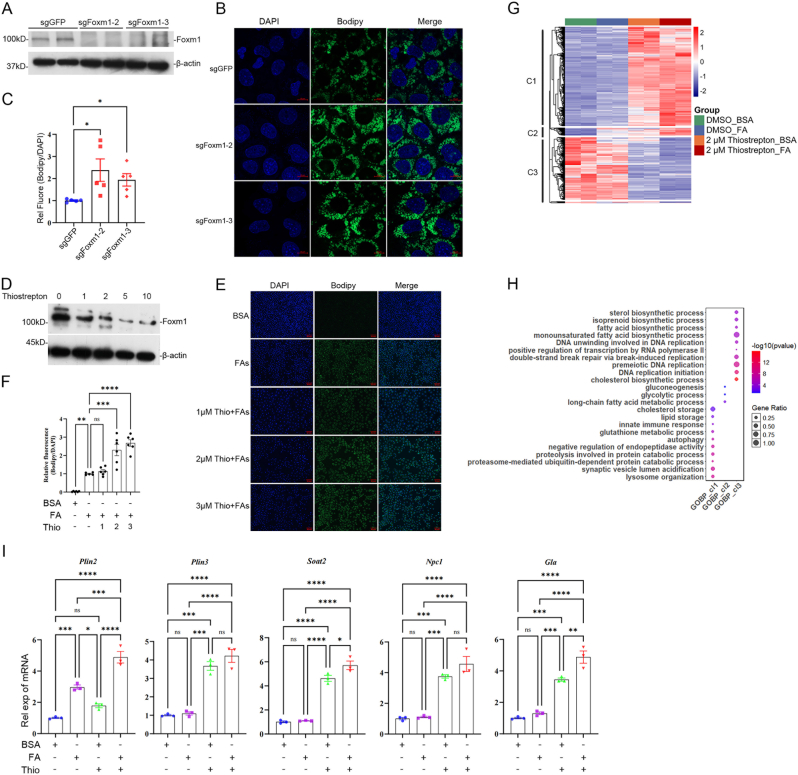
**Foxm1 regulates transcriptional programs of lipid metabolisms in liver cells. (A)** Western blot validation of Foxm1 protein levels in AML12 knockout cell lines. **(B)** Fluorescence staining of sgFoxm1 AML12 knockout cells treated with FAs (oleic acid, 600 μM + palmitic acid, 300 μM). DAPI, blue; Bodipy, green; 63 × oil objective. Scale bar = 10 μm. **(C)** Statistical analysis of relative fluorescence intensity. **(D)** Western blot analysis of Foxm1 protein expression in AML12 cells treated with DMSO, 1 μM, 2 μM, 5 μM, and 10 μM thiostrepton for 24 h. **(E&F)** AML12 were treated with thiostrepton at the indicated concentrations together with FAs. Lipid droplets were stained with Bodipy. **(G)** Heatmap displaying DEGs across four treatment groups: BSA, FAs, 2 μM thiostrepton + BSA, and 2 μM thiostrepton + FAs, categorized into three distinct clusters (Clusters 1-3). **(H)** GO functional analysis of the three gene expression clusters. **(I)** RT-qPCR validation of mRNA expression for *Plin2*, *Plin3*, *Soat2*, *Gla*, and *Npc1*. ∗*P* < 0.05, ∗∗*P* < 0.01, ∗∗∗*P* < 0.001, ∗∗∗∗*P* < 0.000 1; ns indicates not significant.

To investigate whether Foxm1 regulates lipid metabolism in liver cells, we constructed *Foxm1* knockout cell lines in AML12 cells (Fig. 6(A)). Bodipy staining showed that the amount of lipid droplets in cytosol significantly increased in knockout cell lines (Fig. 6(B) and (C)). The results further supported by two distinct inhibitors. Thiostrepton is a small chemical inhibitor for Foxm1 (Bhat et al., 2009). The protein level of Foxm1 in AML12 decreased after thiostrepton treatment in a dose-dependent manner (Fig. 6(D)). Bodipy staining showed that thiostrepton treatment increased the amount of lipid droplets at a dose dependent manner, both in AML12 and HepG2 cells (Fig. 6(E) and (F), Fig. S6A). FDI-6 is another small chemical inhibitor by directly binding to Foxm1 (Gormally et al., 2014). It also increased lipid droplets in the above two cell lines (Fig. S6B and C). These indicate that Foxm1 suppresses lipid droplet accumulation in liver cells.

### 2.9 Foxm1 represses the gene expression of lipid metabolism

To investigate the mechanism for Foxm1 in lipid metabolism regulation, AML12 cells were treated with thiostrepton with or without fatty acids (oleic acid, 600 μM + palmitic acid, 300 μM), and then analyzed by RNA-Seq. The results showed that thiostrepton enhanced the expression of genes enriched in lipid storage, cholesterol metabolism, autophagy, innate immune response, and glycolysis pathway, and repressed the genes enriched in lipid biosynthetic processes and DNA replication (Fig. 6(G) and (H), Table S7). Several upregulated genes were verified using qRT-PCR, including lipid droplet-associated proteins (*Plin2*, *Plin3*, *Plin5*), cholesterol esterification enzymes (*Soat1*, *Soat2*), lysosomal enzymes (*Hexa*, *Hexb*, *Gla*, *Gm2a*), and cholesterol transport protein (*Npc1*) (Fig. 6(I), Fig. S6D). These indicate that Foxm1 represses hepatic lipid accumulation in MASLD by regulating the expression of lipid metabolic genes.

## 3 Discussion

This study constructed a comprehensive epigenetic atlas of MASLD progression using a GAN diet-induced MASLD mouse model. It revealed dynamic shifts in gene expression from metabolic imbalance to inflammatory activation and fibrotic changes, characterized by upregulation of inflammatory responses and downregulation of lipid metabolism. Histone modifications exhibited dynamic enrichment and interconversion at promoter/enhancer regions, with H3K27ac and H3K9me3 playing key roles in lipid metabolic dysregulation, immune activation, and fibrosis. ChromHMM analysis uncovered significant chromatin state remodeling, particularly in enhancer regions, while increased heterochromatin proportion reflected progressive epigenetic changes. Motif analysis identified nine transcription factors, and functional studies demonstrated that Foxm1 suppresses hepatic lipid accumulation by regulating lipid and cholesterol storage pathways. These findings highlight the central role of epigenetic regulation in MASLD progression.

Epigenetic factors dynamically regulate gene expression during MASLD progression, influencing key pathological processes such as lipid metabolism, inflammatory responses, and fibrogenesis. Tracking temporal epigenomic changes is critical for understanding MASLD pathogenesis. Epigenetic modifications undergo dynamic alterations across disease stages. Monitoring multiple time points allows us to capture the full spectrum of these changes and elucidate their roles in disease initiation, progression, and potential regression. Understanding of epigenetic changes during critical time windows can guide the selection of optimal intervention strategies, such as drugs targeting epigenetic enzymes or gene-editing approaches, thereby enabling precise and stage-specific treatments. The current study established the first comprehensive epigenetic atlas of MASLD by integrating both MCD diet-induced MASH and GAN diet-induced full-spectrum disease models across multiple disease stages, systematically deciphering the epigenetic regulatory mechanisms underlying disease progression. We used ChromHMM, a computational tool that leverages multiple histone modification signals, for systematic chromatin state annotation (Ernst and Kellis, 2017). This approach revealed stage-specific changes in enhancer, promoter, heterochromatin, and other chromatin states during MASLD pathogenesis, thereby elucidating underlying gene regulatory mechanisms. Chromatin state analysis has demonstrated the interconversion between chromatin states across disease stages, indicating that MASLD progression does not follow a linear trajectory but rather manifests as a multi-stage dynamic continuum involving regulated transitions between distinct phenotypic states.

Enhancers, as critical cis-regulatory elements, modulate gene transcription through the formation of chromatin loop structures, affecting hepatic lipid accumulation, insulin resistance, and fibrosis (Li et al., 2024; Ma et al., 2022). In the GAN diet model, the number of enhancers increased significantly during disease progression. Temporal analysis identified six distinct dynamic patterns: Clusters 1 and 4 (decreasing) were associated with lipid metabolism; clusters 2 and 5 (increasing) correlated with inflammatory responses; and clusters 3 and 6 (increasing) were linked to fibrotic processes, indicating stage-specific regulatory functions of enhancers. Our study systematically elucidates the central role of epigenetic regulation in MASLD. Future work should delineate promoter activation and silencing patterns of critical metabolic, inflammatory, and fibrosis-related genes throughout disease progression. Determining the existence of stage-specific promoters or repressors as diagnostic biomarkers will be valuable. Applying single-cell epigenomics will resolve cell type-specific chromatin states. Leveraging targeted epigenetic tools including CRISPR-dCas9 systems will advance novel MASLD therapy development.

Transcription factors (TFs) are key regulators of cellular homeostasis and disease states. The forkhead box (FOX) protein family, through its DNA-binding domains, participates in processes such as glucose/lipid metabolism, immune regulation, and disease pathogenesis. These functions are primarily achieved through transcriptional regulation and interactions with cofactors such as MuvB and STAT3 (Zhang et al., 2023a). Foxm1, a member of the FOX family, plays important roles in cell cycle regulation, DNA repair, metabolism, and carcinogenesis (Kang et al., 2020; Ziman et al., 2024). Previous studies have demonstrated its involvement in diabetes, liver fibrosis, and HCC (Filliol and Schwabe, 2020; Golson et al., 2010; Yang et al., 2024), where it promotes liver regeneration (Izumi et al., 2018) and is associated with HCC proliferation and invasion (Hu et al., 2020). In bile duct ligation models, Foxm1 deficiency alleviates inflammation and fibrosis (Yang et al., 2024). However, its role in MASLD lipid metabolism remains unclear. This study found significantly reduced Foxm1 protein levels in both MCD and GAN diet-induced MASLD models. *In vivo* and *in vitro* experiments consistently demonstrated that Foxm1 inhibition promotes lipid accumulation, contradicting previous findings (Ma et al., 2025b). Treatment of AML12 cells with the Foxm1 inhibitor thiostrepton upregulated genes related to lipid/cholesterol storage (Plin2/3/5, Soat1/2, Npc1) and lysosomal function (Hexa/b, Gla, Gm2a), indicating that Foxm1 acts to suppress MASLD progression by regulating lipid storage pathways. Future studies should identify target genes through ChIP-Seq and further investigate its regulatory mechanisms in human MASLD. However, this study is limited and further investigations need to be carried out in the future. First, although we have demonstrated the role of Foxm1 in lipid metabolism, the lack of Foxm1 ChIP-Seq data to directly identify its downstream target genes prevents a comprehensive understanding of Foxm1 transcriptional network. Future studies should optimize ChIP experimental conditions or employ other technologies such as CUT&Tag to capture Foxm1 binding sites directly in hepatocytes or liver tissues. Second, the functional study relied on small molecule inhibitors of Foxm1. Although we selected two inhibitors with relatively high specificity, the possibility of off-target effects cannot be completely excluded. Future studies should incorporate genetic approaches, such as hepatocyte-specific *Foxm1* knockout mice, to further validate the function of Foxm1 both *in vivo* and *in vitro*. Additionally, although Foxm1 protein is downregulated during MASLD progression, its transcriptional level is elevated, and the underlying mechanisms remain unclear. Subsequent studies should investigate the roles of post-translational modifications, protein stability regulation, and cell type-specific expression in the process.

In summary, this study constructed an epigenetic regulatory network for MASLD by integrating transcriptomic and epigenomic data. Through this network, we revealed extensive remodeling of H3K27ac, H3K4me1, H3K4me3, H3K9me3, and H3K27me3 modifications during MASLD progression, and for the first time identified Foxm1 involvement in MASLD pathogenesis. These findings provide novel potential targets for early diagnosis, disease course management, and targeted therapy of MASLD.

## 4 Methods and materials

### 4.1 Animal housing

The C57BL/6J male mice were purchased from GemPharmatech Co., Ltd. All mice were maintained under specific pathogen-free (SPF) conditions at 20–24 °C with humidity of 40%–70% and a 12-h light/dark cycle (lights on at 7:00 a.m., lights off at 7:00 p.m.), with free access to water and food (Animal Center of College of Life Sciences, Wuhan University).

### 4.2 Reagents and cell lines

Antibodies were purchased from the manufacturers and their information is included in Table S1. The PCR primers were synthesized by Tsingke Biotechnology Co., Ltd., and the sgRNA primers were synthesized by Youkang Biotechnology Co., Ltd (Table S1). AML12 and HepG2 cell lines were purchased from Cell Bank of Chinese Academy of Sciences (Shanghai, China) and cultured under recommended conditions according to the manufacturer's instructions with 10% FBS.

### 4.3 Construction of the MASLD mouse model

Forty male C57BL/6J mice (5 weeks old, approximately 20 g body weight) (GemPharmatech Co., Ltd., Jiangsu, China) were randomly assigned to a control group (*n* = 16) and a GAN group (*n* = 24). After one week of acclimatization, the control group was fed a standard maintenance diet (Wuhan Qianjiajiaxing Biotechnology Co., Ltd., China), while the GAN group received a high-fat, high-cholesterol, high-fructose diet (Research Diets, D09100310). Mice were sacrificed at 8 weeks (control, *n* = 5; GAN, *n* = 5), 16 weeks (control, *n* = 5; GAN, *n* = 6), 20 weeks (control, *n* = 3; GAN, *n* = 5), and 28 weeks (control, *n* = 3; GAN, *n* = 8). To study the function of Foxm1, we induced an MASLD model using a methionine and choline deficiency diet (MCD, Research Diets, A02082002B) for 4 weeks. Mice were fasted for 6–8 h prior to sacrifice but had free access to water. Whole blood was collected via retro-orbital bleeding, after which the animals were immediately sacrificed by cervical dislocation. Liver tissues were then collected following perfusion. Both serum and liver samples were stored at −80 °C for subsequent analysis.

### 4.4 Serological testing

Fresh whole blood samples were collected into standard microcentrifuge tubes and allowed to clot overnight at 4 °C. The clotted samples were then centrifuged at 3 000 rpm for 10 min at 4 °C. The supernatant (serum) was carefully aspirated and used for subsequent analyses. Serum alanine aminotransferase (ALT) and aspartate aminotransferase (AST) activities were measured using the Alanine Aminotransferase (ALT/GPT) Assay Kit (Nanjing Jiancheng Bioengineering Institute, Cat. No. C009-2-1) and the Aspartate Aminotransferase (AST/GOT) Assay Kit (Nanjing Jiancheng Bioengineering Institute, Cat. No. C010-2-1), respectively. Serum total cholesterol (TC) and triglyceride (TG) levels were determined using the Total Cholesterol (T-CHO) Assay Kit (Nanjing Jiancheng Bioengineering Institute, Cat. No. A111-1-1) and the Triglyceride (TG) Assay Kit (Nanjing Jiancheng Bioengineering Institute, Cat. No. A110-1-1), respectively.

### 4.5 Hepatic TC and TG measurement

Liver tissue was weighed and homogenized in absolute ethanol at a 1:9 (*W*/*V*) ratio, followed by centrifugation at 2 500 rpm for 10 min at 4 °C to collect the supernatant. Hepatic total cholesterol (TC) and triglyceride (TG) levels were quantified using commercial assay kits (Total Cholesterol Kit, Cat# A111-1-1; Triglyceride Kit, Cat# A110-1-1, Nanjing Jiancheng Bioengineering Institute) according to the manufacturer protocols.

### 4.6 Oral glucose tolerance test (OGTT)

Mice were transferred to clean cages and fasted for >6 h with free access to water. After recording body weights, mice were restrained in holders and their tails were sterilized with 75% ethanol. The distal 1–2 mm tail tip was excised, and the first blood drop was discarded. Fasting blood glucose (0 min baseline) was measured from the second drop using a glucometer. Mice received oral gavage of 2 g/kg glucose (200 mg/mL in saline) at 0.01 mL/g body weight. Blood glucose levels were measured at 30, 60, and 120 min post-gavage, with individual procedures staggered by 1–3 min. Post-test, mice were returned to cages with fresh feed for data analysis.

### 4.7 Bodipy staining

Cells in 24-well plates were fixed with 4% paraformaldehyde at room temperature for 20 min, washed three times with PBS, and incubated with 10 μM Bodipy (MedChemExpress, Cat# HY-D1614) in PBS for 40 min at room temperature in the dark. After three PBS washes, nuclei were counterstained with 5 μg/mL DAPI (Merck, Cat# D9542) in PBS for 10 min in the dark. After final PBS washes, lipid droplets were visualized using fluorescence microscopy.

### 4.8 H&E staining

Fresh tissues were fixed in 4% paraformaldehyde, followed by paraffin embedding and sectioning. Sections were sequentially immersed in Xylene I for 20 min, Xylene II for 20 min, Absolute Ethanol I for 5 min, Absolute Ethanol II for 5 min, 75% Ethanol for 5 min, and finally rinsed with tap water. Subsequently, sections were stained in Hematoxylin solution for 3-5 min, differentiated using a differentiation solution, treated with a bluing solution, and rinsed under running water. Then, sections were dehydrated sequentially in 85% and 95% graded ethanol (5 min each), followed by staining in Eosin solution for 5 min. Finally, sections were dehydrated and cleared by sequential immersion in Absolute Ethanol I for 5 min, Absolute Ethanol II for 5 min, Absolute Ethanol III for 5 min, Xylene I for 5 min, Xylene II for 5 min, and mounted with neutral gum.

### 4.9 Masson staining

Fresh tissues were fixed in 4% paraformaldehyde, followed by paraffin embedding and sectioning. Sections were sequentially immersed in Xylene I (20 min) - Xylene II (20 min) - Absolute Ethanol I (10 min) - Absolute Ethanol II (10 min) - 95% Ethanol (5 min) - 90% Ethanol (5 min) - 80% Ethanol (5 min) - 70% Ethanol (5 min), and finally rinsed with tap water. Subsequently, sections were stained with Weigert's Iron Hematoxylin solution from the Masson staining kit for 5 min, differentiated in 1% hydrochloric acid alcohol for several seconds, rinsed under running water, and blued. Next, sections were stained in Ponceau Acid Fuchsin solution for 5-10 min, treated with phosphomolybdic acid aqueous solution for 3-5 min, followed by treatment with 1% glacial acetic acid for 1 min. Finally, sections were dehydrated and cleared by sequential immersion in 95% Ethanol I (5 min) - 95% Ethanol II (5 min) - Absolute Ethanol I (5 min) - Absolute Ethanol II (5 min) - Xylene I (5 min) - Xylene II (5 min), and mounted with neutral gum.

### 4.10 Oil Red O staining

Fresh tissues were fixed in 4% paraformaldehyde and processed for frozen sectioning. Sections were stained in freshly prepared Oil Red O working solution for 7-10 min (protected from light), differentiated in Differentiation Solution B for 5-10 s, and rinsed twice with tap water. Subsequently, sections were stained with Hematoxylin Solution C for 2-4 min, differentiated in Hematoxylin Differentiation Solution D for 2-3 s, blued in Hematoxylin Bluing Solution E for 2-3 s, rinsed with tap water, and mounted with neutral gum.

### 4.11 RNA-seq library construction

Approximately 10 mg of liver tissues were homogenized and collected by centrifugation. Total RNA was extracted with an RNA extraction kit (CWBIO, CW0581) according to the manufacturer's instruction. Next, approximately 1 μg of total RNA was used for RNA enrichment kit (Abclonal, RK20340) to obtain mRNA. Then, cDNA synthesis, adaptor addition and library amplification processes were performed according to Fast RNA-seq Lib Prep Kit V2 protocol (Abclonal, RK20306). Finally, the library was sequenced in DNBSEQ-T7 platform after quality test in Agilent 2100.

### 4.12 ChIP-Seq library preparation

A 60 mg sample of mouse liver tissue was fixed with formaldehyde, quenched with glycine, and homogenized using steel beads. Following cell lysis, chromatin was digested with Micrococcal Nuclease at 37 °C for 20 min and fragmented by sonication on ice. The lysate was immunoprecipitated overnight at 4 °C with rotation using Protein G magnetic beads and a specific antibody. The bead complexes were then sequentially washed with different elution buffers. Crosslinks were reversed and DNA was released by incubating with Elution Buffer and Proteinase K at 65 °C. The decrosslinked DNA was purified using a universal DNA purification kit (TIANGEN DP214). Sequencing libraries were constructed from the purified DNA using the VAHTS Universal DNA Library Prep Kit for Illumina V3 (Vazyme ND607-02), which included end repair, adapter ligation, bead-based purification, and PCR amplification steps. The amplified libraries were purified with magnetic beads, quantified using a Qubit fluorometer, and quality-checked by 2% agarose gel electrophoresis. Final sequencing was performed on the Illumina HiSeq X Ten platform.

### 4.13 ChIP-Seq data analysis

Raw ChIP-Seq reads were preprocessed using fastp for adapter trimming and quality filtering (-q 25 -l 36 -f 6 -F 6). The processed reads were then aligned to the mm10 reference genome using Bowtie2 (2.3.5.1, https://github.com/BenLangmead/bowtie2). PCR duplicates were removed using picard MarkDuplicates (2.27.5, https://github.com/broadinstitute/picard) with the option --REMOVE_DUPLICATES true. ChIP-Seq signals were quantified using FPKM (Fragments Per Kilobase per Million mapped reads).

### 4.14 RNA-seq data analysis

Raw sequencing reads were preprocessed by fastp (0.22.0, https://github.com/OpenGene/fastp) for adapter trimming and quality filtering (-q 25 -l 30 -f 10 -F 10). After that, the filtered reads underwent quality assessment with FastQC (v0.12.1, https://github.com/s-andrews/FastQC). Filtered reads were then aligned to the mm10 reference genome using HISAT2 (2.2.1, https://daehwankimlab.github.io/hisat2/). Uniquely mapped reads were quantified with featureCounts (v2.0.1, https://github.com/ShiLab-Bioinformatics/subread). Gene expression levels were quantified using FPKM. DEGs were identified using DESeq2 (1.42.1, https://github.com/thelovelab/DESeq2), with the threshold set at |log_2_FoldChange| ≥ 1 and adjusted *P*-value (*P*adj) < 0.05. To further investigate the biological significance of the DEGs, Gene Set Enrichment Analysis (GSEA) was performed using ClusterProfiler (4.10.1, https://guangchuangyu.github.io/software/clusterProfiler/) with gseGO (ontology: BP), and the results were visualized with GseaVis (0.1.0, https://github.com/junjunlab/GseaVis).

### 4.15 Principal component analysis (PCA) and sample correlation

Sample relationships were assessed using DESeq2 normalized data to evaluate biological consistency and identify potential outliers. For RNA-Seq PCA, variance-stabilized transformed (VST) counts were processed via the plotPCA function, with principal components calculated based on the top 500 most variable genes across all samples, and results were visualized using ggplot2. For ChIP-Seq samples, PCA was performed on the normalized read counts in consensus peak regions across all samples using the R package FactoMineR, enabling assessment of global epigenetic variation between samples and modifications. Spearman correlation coefficients between samples were calculated from normalized expression or peak intensity matrices using the cor() function in R and visualized as heatmaps via the pheatmap package, with hierarchical clustering to reveal sample grouping patterns.

### 4.16 Chromatin state annotation

To systematically characterize combinatorial patterns of histone modifications and define chromatin states across MASLD progression, we used ChromHMM (version 1.24) to train a multivariate hidden Markov model. The mapped BED files of all five histone modification markers (H3K27ac, H3K4me1, H3K4me3, H3K27me3, and H3K9me3) across four MASLD development stages were binned into non-overlapping 200-bp intervals across the entire genome using Bedtools software (version 2.30.0). Input alignment files from matched samples were used as controls to provide background signal levels and adjust the binarization criteria. For binarization, a Poisson *p*-value threshold of 1e-4 was used to determine the presence of each histone modification in each genomic bin, comparing ChIP signal to input control. The model was trained with a range of chromatin states from 10 to 15, evaluating model fit based on reproducibility of state assignments across biological replicates and enrichment patterns on known genomic features such as promoters, gene bodies, and intergenic regions. A 14-state model was ultimately selected for subsequent analysis as it optimally captured the key combinatorial patterns and interactions among all five histone modification markers. Using the emission probabilities of each state and the enrichment of each state on genomic annotation regions calculated by ChromHMM, each chromatin state was manually annotated with a functional label based on its predominant histone modification signature and genomic localization patterns (e.g., active promoter, poised enhancer, polycomb-repressed, heterochromatin). These 14 chromatin states were further classified into five major categories: enhancer regions, promoter regions, transcriptionally repressed regions (polycomb-associated), heterochromatin regions (H3K9me3-associated), and quiescent regions (no significant modification enrichment). ChromHMM annotated every 200-bp genomic interval with a specific chromatin state for each stage of MASLD development, creating a comprehensive chromatin state map across the entire genome and time course.

### 4.17 Quantitative real-time PCR

RNA was extracted from mouse liver tissue and cultured cells using the Ultrapure RNA Kit (CWBIO, CW0581M) and EASYspin Kit (Aidlab, RN07), respectively. Tissue samples were lysed in TRIzon Reagent followed by chloroform phase separation, while cells were directly lysed. RNA was prepared according to the manufacturer's protocol. cDNA was synthesized using ABScript Neo RT Master Mix with gDNA Remover (Abclonal, RK20433). RT-qPCR was performed using 2 × Universal SYBR Green Fast qPCR Mix (Abclonal, RK21203). Relative gene expression levels were measured with quantitative PCR, β-actin as normalization.

### 4.18 Western blot

Liver tissue was homogenized using steel beads in PBS, washed, and lysed in RIPA buffer; lysates were mixed with an equal volume of 2 × SDS Loading Buffer containing β-mercaptoethanol, and denatured at 95 °C for 15 min. Cultured cells were directly lysed in 2 × SDS Loading Buffer. Electrophoresis was performed and proteins were transferred to nitrocellulose membranes via wet transfer. Membranes were washed with TBST, blocked with 5% skim milk/TBST for 1 h, then incubated with primary antibodies at 4 °C overnight, secondary antibodies at room temperature for 30 min, and washed 3 times with TBST. Protein signals were detected using ECL substrate, followed by film development.

### 4.19 Construction of knockout cell lines

Primers for sgRNAs (Table S1) were designed using the Broad Institute CRISPR Design Platform (https://portals.broadinstitute.org/gppx/crispick/public). Plasmids containing sgRNA fragments were constructed from pLentiCRISPR v2 vector. The verified lentiviral plasmids were co-transfected with psPAX2 and pMD2.G packaging plasmids at a 1:1:1 ratio into 293T cells via calcium phosphate transfection. Lentiviral supernatant was collected 48-72 h post-transfection, filtered through 0.45 μm membranes, and used to infect target cells with 1:1 000 polybrene. Puromycin selection was initiated after 48 h of infection, with final knockout efficiency confirmed by quantitative real-time PCR (RT-qPCR) and western blot analysis.

### 4.20 Establishment of liver knockout mouse models

To establish knockout mouse livers, C57BL/6J male mice (5 weeks old, approximately 20 g body weight) were randomly administered with sgFoxm1 plasmid or empty vector via hydrodynamic injection after one week of acclimatization. Specifically, a total of 50 μg of plasmid DNA was dissolved in sterile saline solution, and the injection volume (mL) was set to 10% of the mouse body weight. The injection was completed rapidly within 5–7 s, after which the mice were placed in a warm environment for recovery. One-week post-injection, plasmid-treated mice were further randomized into normal diet and MCD diet groups and euthanized after 4 weeks of dietary intervention.

### Ethics approval

All the animal operations followed the laboratory animal guidelines of Wuhan University and were approved by the Animal Experimentations Ethics Committee of Wuhan University (Protocol NO. 14110B).

### Statistical analysis

Statistical analyses in this study were performed using GraphPad Prism software. Each experimental group contained at least three technical or biological replicates. Data are presented as mean ± standard error of the mean (mean ± SEM). Statistical significance between groups was determined using Student's *t*-test (two-tailed). Significance levels are denoted as follows: ∗*P* < 0.05, ∗∗*P* < 0.01, ∗∗∗*P* < 0.001, ∗∗∗∗*P* < 0.000 1, ns indicates not significant.

## CRediT authorship contribution statement

**Chuanfei Zeng:** Investigation. **Mingliang Wei:** Formal analysis. **Huan Li:** Investigation. **Fengyuan Niu:** Resources. **Ziqing Guo:** Resources. **Lian-Yun Li:** Supervision, Project administration. **Min Wu:** Writing – review & editing, Writing – original draft, Supervision, Project administration, Funding acquisition, Conceptualization. **Ming-Kai Chen:** Writing – review & editing, Supervision, Project administration, Funding acquisition.

## Declaration of competing interest

The authors declare that they have no known competing financial interests or personal relationships that could have appeared to influence the work reported in this paper.

## Data Availability

The raw sequence data reported in this paper have been deposited in the Genome Sequence Archive (Zhang et al., 2025) in National Genomics Data Center (Members C.-N. and Partners, 2025), China National Center for Bioinformation/Beijing Institute of Genomics, Chinese Academy of Sciences (GSA: CRA033335) that are publicly accessible at https://ngdc.cncb.ac.cn/gsa.
