# Supplementary material for: Identification of Foxm1 as a critical regulator for metabolic dysfunction-associated steatotic liver disease by epigenomic and transcriptional profiling
Source: Cell Insight. 2026 Apr 14;5(3):100325. doi: 10.1016/j.cellin.2026.100325 (PMC13137179; doi:10.1016/j.cellin.2026.100325)
Supplement: Multimedia component 1 [file mmc1.pdf]

Sup Figure S1

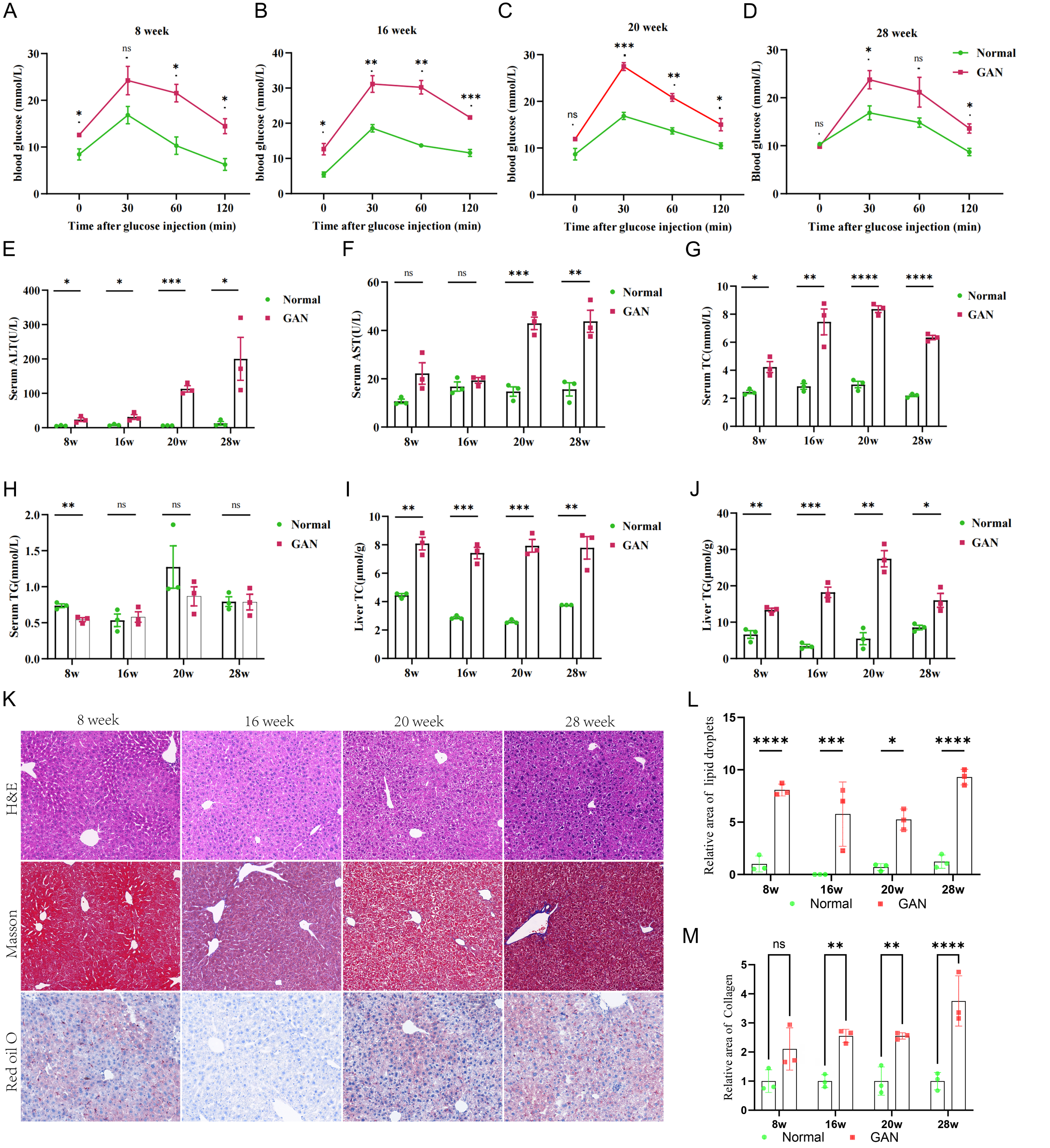

Sup Figure S2

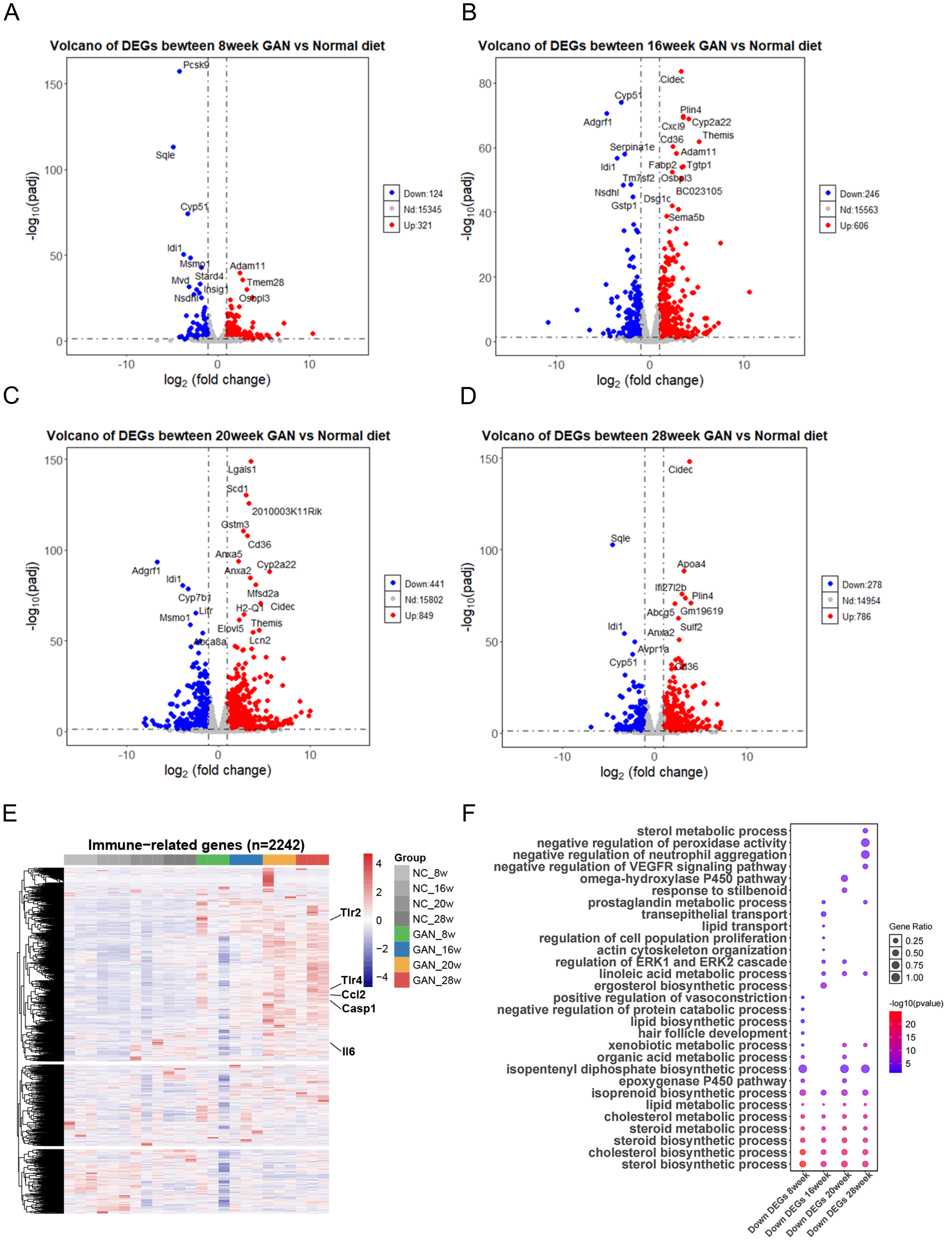

Sup Figure S3

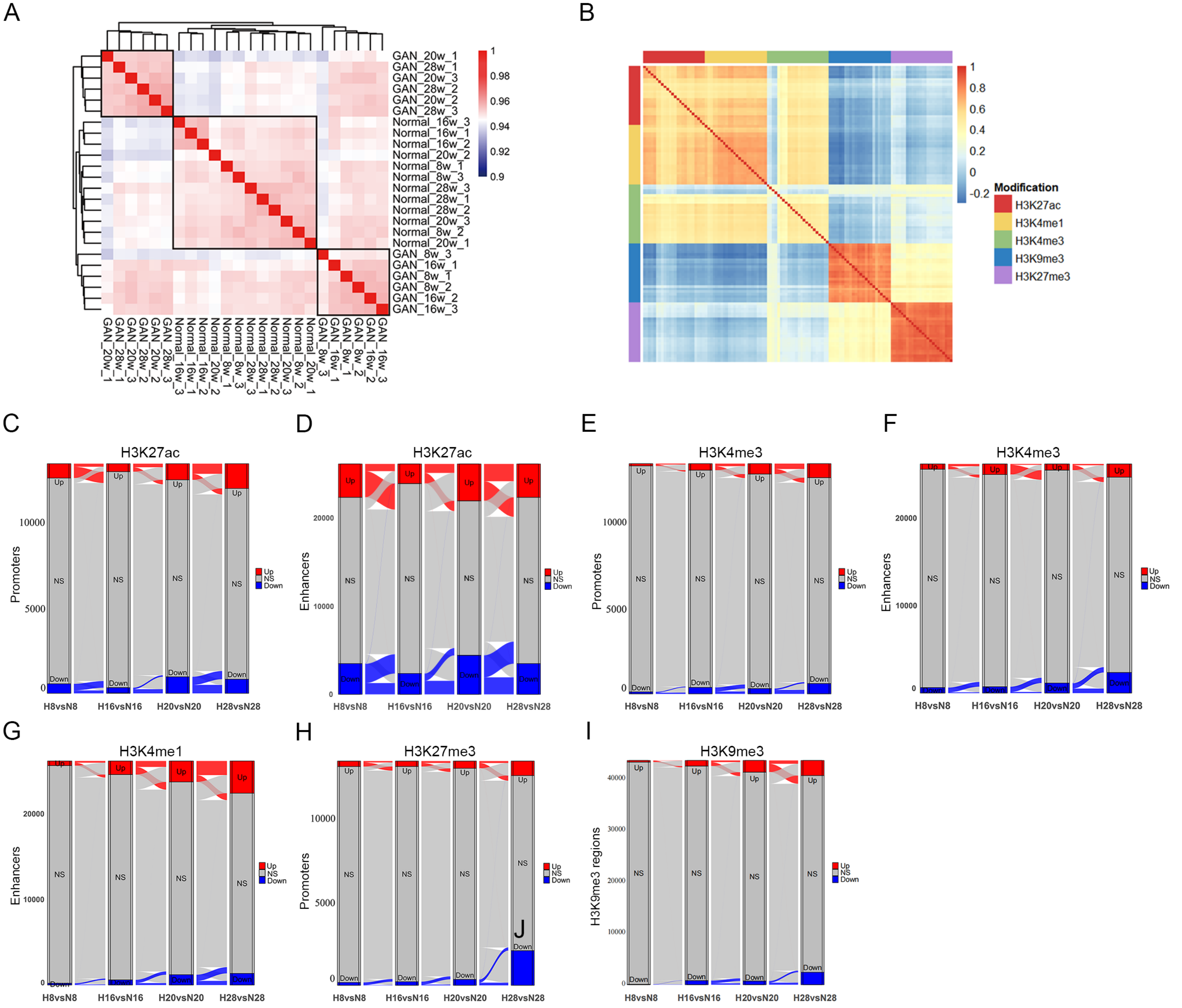

Sup Figure S4

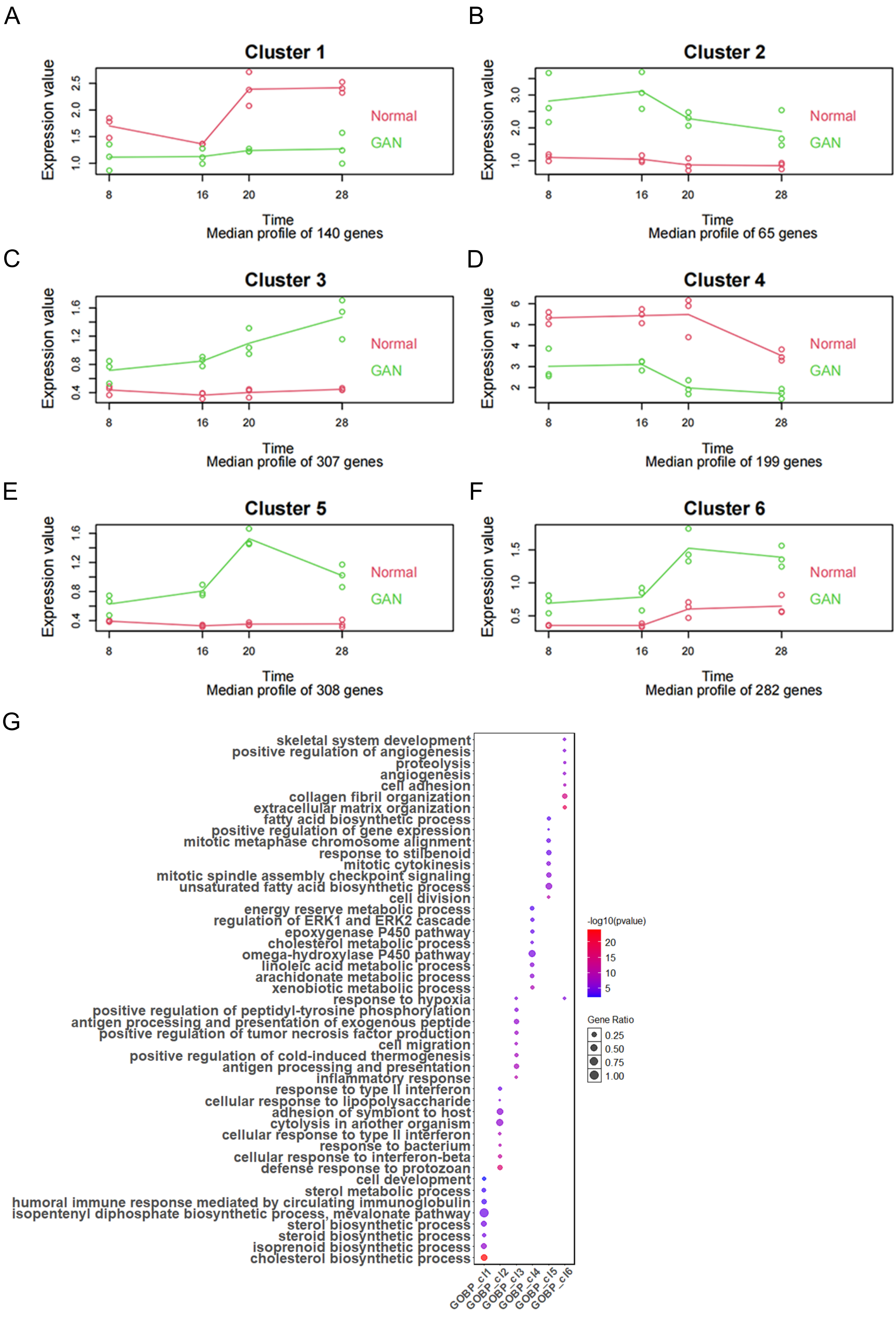

Sup Figure S5

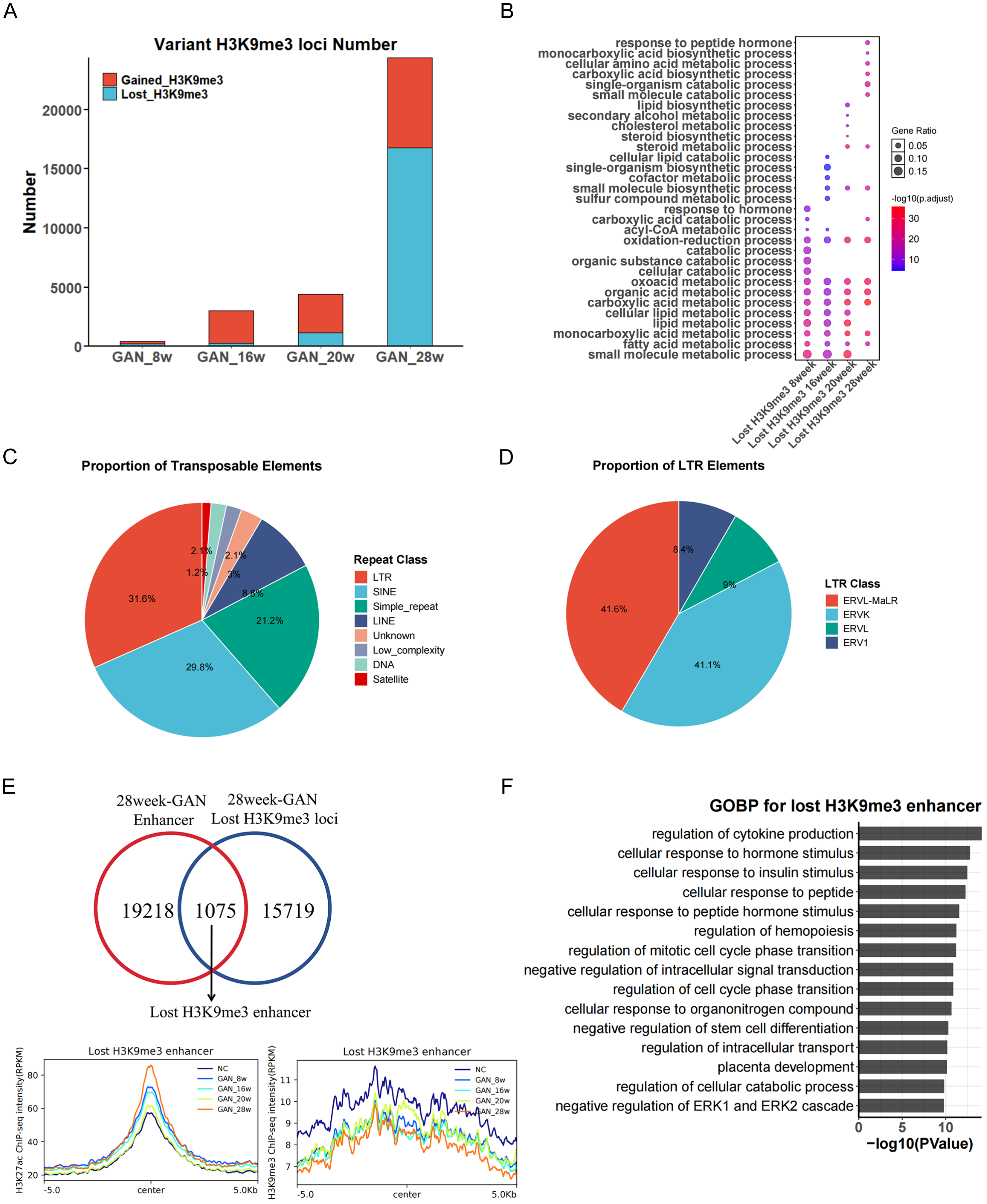

Sup Figure S6

A

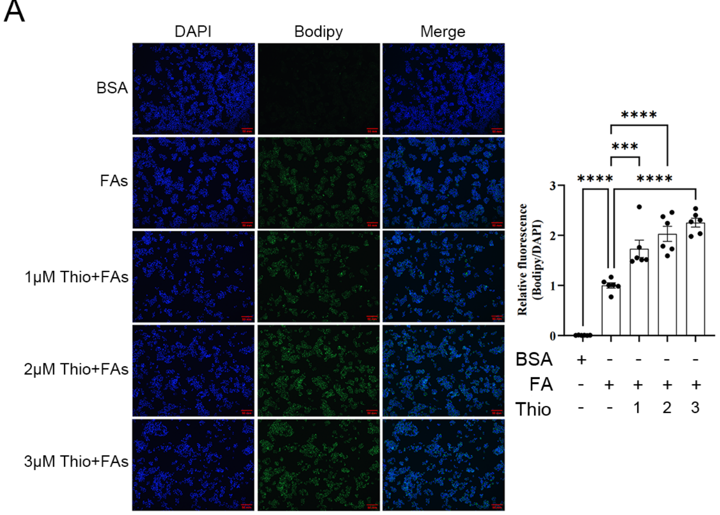

B

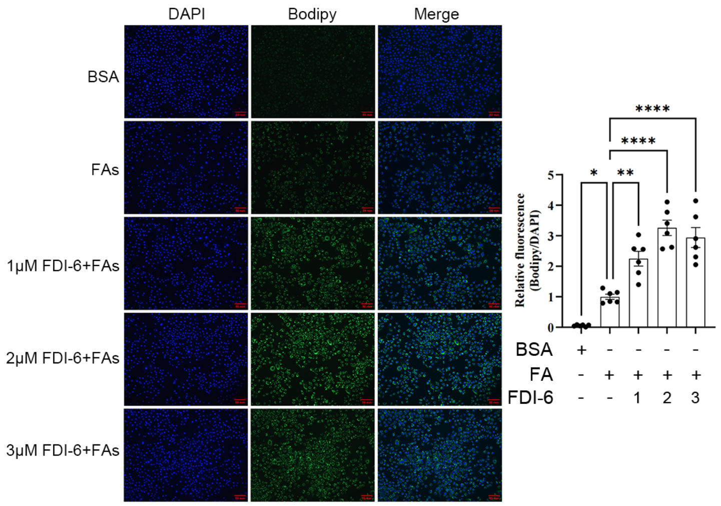

C

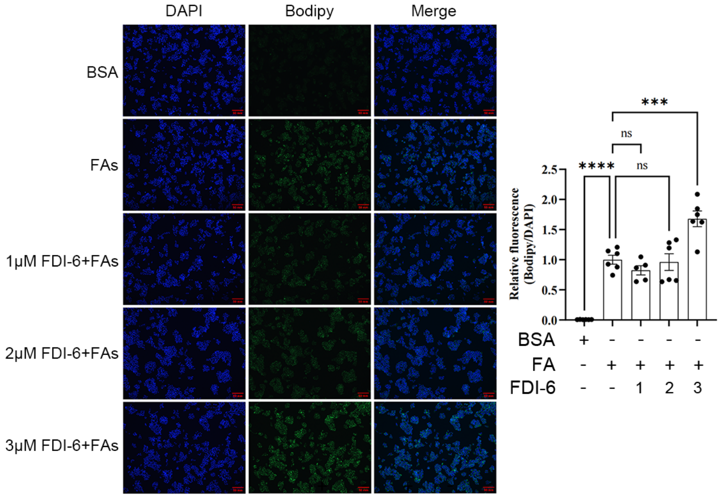

D

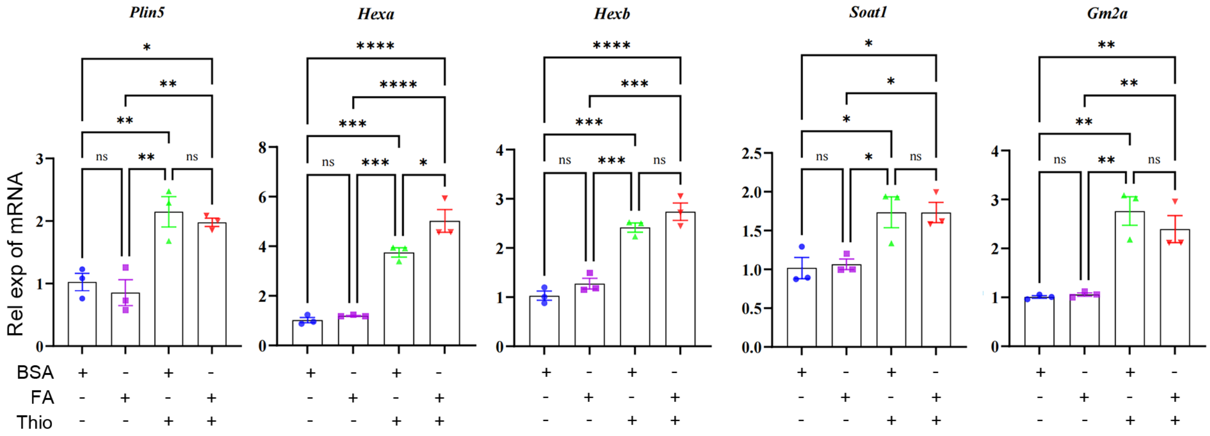

Supplemental figure legends

Sup. Fig. 1. Metabolic parameter profiles in GAN diet-induced MASLD mice across timepoints. (A-D) Fasting blood glucose levels in 8-week (A), 16-week (B), 20-week (C) and 28-week (D) mice. The GAN group was compared with the Normal group at each corresponding time point (0, 30, 60, and 120 minutes). **(E-J)** Serum ALT (E), Serum AST levels (F), Serum TC levels (G), Serum TG levels (H), Hepatic TC contents (I), and Hepatic TG contents (J) at 8, 16, 20, and 28 weeks in GAN diet-induced mouse livers. **(K)** H&E, Masson, and Oil Red O staining images of livers from normal diet-fed control mice at the indicated time points. **(L&M)** Quantification of lipid droplets (L) and relative area of collagens (M) in the animal livers. *, $P < 0.05$; **, $P < 0.01$; ***, $P < 0.001$; ****, $P < 0.0001$; ns, not significant.

Sup. Fig. 2. Temporal dynamics of transcriptomic program in MASLD progression. (A-D) Volcano plots of differentially expressed genes (DEGs) between GAN and Normal groups at 8 weeks (A), 16 weeks (B), 20 weeks (C), and 28 weeks (D). The X-axis represents the $\log_2$ fold change ($\log_2FC$) in gene expression, and the Y-axis represents $-\log_{10}(Padj)$, indicating statistical significance. Red dots denote significantly upregulated genes ($\log_2FC >$ threshold, $P < 0.05$), blue dots denote significantly downregulated genes ($\log_2FC <$ -threshold, $P < 0.05$), and gray dots denote non-significant genes ($P \geq 0.05$). Dashed lines indicate thresholds for statistical significance (fold change and P-value). **(E)** Heat maps of immune-related DEGs in the livers of MASLD mice at 8, 16, 20, and 28 weeks. **(F)** GO functional analysis of down-regulated DEGs in the livers of MASLD mice at 8, 16, 20, and 28 weeks.

Sup. Fig. 3. Dynamics chromatin landscapes of histone modifications during MASLD progression. (A) Correlation heatmap between RNA-Seq sample. **(B)** Correlation heatmap between ChIP-Seq samples. **(C)** Changes in H3K27ac modification in promoter regions of the liver in MASLD mice at 8, 16, 20, and 28 weeks. **(D)** Changes in H3K27ac modification in enhancer regions. **(E)** Changes in

H3K4me3 modification in promoter regions. **(F)** Changes in H3K4me3 modification in enhancer regions. **(G)** Changes in H3K4me1 modification in enhancer regions. **(H)** Changes of H3K27me3 regions during MASLD progression. **(I)** Changes of H3K9me3 regions during MASLD progression.

Sup. Fig. 4. The temporal dynamics of transcriptional programs during MASLD progression. (A-F) MaSigPro time-series clustering of significant DEGs identified six clusters. **(G)** GO analysis of the above 6 clusters.

Sup. Fig. 5. The dynamics of enhancer landscapes in H3K9me3 regions of MASLD livers. (A) The numbers of the gain and lost H3K9me3 loci in livers of MASLD mice at 8, 16, 20, and 28 weeks. **(B)** Gene Ontology (GO) functional analysis of genes proximal to depleted H3K9me3 regions. **(C)** Proportion of transposable elements annotated at lost H3K9me3 loci in GAN_28w mice. **(D)** Detailed proportion of LTR subclasses within the lost H3K9me3 loci. **(E)** Integration analysis between enhancers and depleted H3K9me3 sites in livers of 28-week GAN diet-induced MASLD mice, showing average H3K9me3 and H3K27ac modification densities at enhancer regions with depleted H3K9me3 marks. **(F)** GO functional analysis of genes proximal to enhancers with depleted H3K9me3 modifications.

Sup. Fig. 6. Foxm1 regulates lipid metabolic program in liver cells. (A) HepG2 were treated with Thiostrepton at the indicated concentrations together with FAs. Lipid droplets were stained with Bodipy. **(B&C)** AML12 (B) or HepG2 (C) cells treated with FDI-6 at the indicated concentrations together with FAs. Lipid droplets were stained with Bodipy. **(D)** RT-qPCR validation of mRNA expression for *Plin5*, *Hexa*, *Hexb*, *Soat1*, *Gm2a*. *$P < 0.05$, **$P < 0.01$, ***$P < 0.001$, ****$P < 0.0001$; ns indicates not significant.
